# Anomaly Detection for Power Quality Analysis Using Smart Metering Systems

**DOI:** 10.3390/s24175807

**Published:** 2024-09-06

**Authors:** Gabriele Patrizi, Cristian Garzon Alfonso, Leandro Calandroni, Alessandro Bartolini, Carlos Iturrino Garcia, Libero Paolucci, Francesco Grasso, Lorenzo Ciani

**Affiliations:** Department of Information Engineering, University of Florence, Via di Santa Marta, 3, 50139 Florence, Italy; cristiancamilo.garzonalfonso@unifi.it (C.G.A.); leandro.calandroni@unifi.it (L.C.); a.bartolini@unifi.it (A.B.); carlos.iturrinogarcia@unifi.it (C.I.G.); libero.paolucci@unifi.it (L.P.); francesco.grasso@unifi.it (F.G.); lorenzo.ciani@unifi.it (L.C.)

**Keywords:** anomaly detection, power quality, machine learning, fault detection, metering systems

## Abstract

The problem of Power Quality analysis is becoming crucial to ensuring the proper functioning of complex systems and big plants. In this regard, it is essential to rapidly detect anomalies in voltage and current signals to ensure a prompt response and maximize the system’s availability with the minimum maintenance cost. In this paper, anomaly detection algorithms based on machine learning, such as One Class Support Vector Machine (OCSVM), Isolation Forest (IF), and Angle-Based Outlier Detection (ABOD), are used as a first tool for rapid and effective clustering of the measured voltage and current signals directly on-line on the sensing unit. If the proposed anomaly detection algorithm detects an anomaly, further investigations using suitable classification algorithms are required. The main advantage of the proposed solution is the ability to rapidly and efficiently detect different types of anomalies with low computational complexity, allowing the implementation of the algorithm directly on the sensor node used for signal acquisition. A suitable experimental platform has been established to evaluate the advantages of the proposed method. All the different models were tested using a consistent set of hyperparameters and an output dataset generated from the principal component analysis technique. The best results achieved included models reaching 100% recall and a 92% F1 score.

## 1. Introduction

Power Quality (PQ) is a critical aspect of modern electrical systems, as it directly affects the performance, reliability, and lifespan of electrical devices and systems. With the increasing integration of renewable energy sources, electric vehicles, and sophisticated electronic loads into the power grid, ensuring robust PQ has become more challenging yet crucial [1,2]. PQ serves as a critical metric for assessing the stability and dependability of electrical power supply, particularly in relation to voltage, current, frequency, and waveform characteristics [3]. Ensuring high PQ is of extreme importance to protect electrical equipment from potential damage, guaranteeing the smooth operation of devices, as well as minimizing energy wastage [4].

Closely associated with the concept of PQ are Power Quality Disturbances (PQDs), which pertain to any irregularities or interruptions that occur in the typical attributes of electrical supply. Such disturbances can adversely affect the overall PQ. Examples of these disturbances encompass voltage surges, sags, transients, harmonics, fluctuations in frequency, and outright interruptions. The origins of PQDs can vary widely, stemming from incidents such as lightning strikes, malfunctions of equipment, faults within the power system, or imbalances in the electrical distribution network. The impacts of these disturbances can be significant, potentially leading to damage or operational failures of electrical equipment, posing safety risks as well [5]. Therefore, maintaining optimal PQ is essential for ensuring the reliable and efficient functioning of electrical systems across residential, commercial, and industrial environments [6].

Nowadays, PQDs have a significant impact across a wide range of industrial sectors. For instance, in [7], the authors conduct a systematic review focused on the various research challenges associated with the real-time identification and classification of PQDs specifically within microgrids. The authors emphasize the critical need for precise detection and categorization of these disturbances to guarantee the dependable and efficient functioning of microgrid systems. Within this review, several primary challenges are identified, which include the necessity for access to high-quality data, the creation of effective methods for feature extraction, the selection of appropriate classification algorithms, and the formulation of robust systems for making decisions. Furthermore, the paper explores a variety of strategies that have been proposed in the academic literature to tackle these identified challenges, while also offering suggestions for future research avenues.

Recent advancements in sensor technologies have opened new avenues for improving PQ monitoring, management, and analysis [8,9]. These sensors, capable of real-time data acquisition and analysis, facilitate the detection of the PQDs [10]. Also, these sensors not only allow for real-time monitoring but also facilitate predictive maintenance and automated corrective actions. Moreover, the deployment of smart sensors in distributed energy systems has shown a promising outcome in enhancing the overall PQ while optimizing energy consumption. As the digitalization of the power sector accelerates, the synergy between PQ management and evolved sensor technologies becomes increasingly significant [11,12].

To analyze the PQ in each system or operating environment, it is necessary to use high quality sensors, that need to be characterized by the following:

- High sensitivity, having the ability to detect small changes in the measured quantity.
- High accuracy, reflecting the lowest possible errors in the voltage and current measurements.
- High precision, providing consistently the same reading for the same input.
- High stability, maintaining performance over time under constant environmental conditions.
- Optimized power consumption, which is critical for energy-efficient applications in the field of smart grids.

Emerging methodologies, including Machine Learning (ML) and Artificial Intelligence (AI), are also being leveraged to analyze the vast amounts of data generated by the sensors, enabling more insightful diagnostics and enhanced decision-making processes. For instance, a novel method based on dictionary learning sparse decomposition is presented in [13], while a robust Discrete Wavelet Transform method integrated with Fast Fourier Transform (FFT) is shown in [14]. Another ML method that uses frequency domain has been previously presented in [15]. Among all ML-based approaches for PQD detection and classification, many researchers are moving toward deep learning [16]. Examples of such deep learning methods are convolutional autoencoders [17] and Long Short-Term Memory (LSTM) networks [18,19]. Another example is convolutional neural networks, which are extremely common in PQD detection, such as the ones published in [20] or [21].

Another important role in tackling the problem of PQ analysis could be played by wireless sensor networks [10,22]. When complex power plants or large manufacturing industries need to be monitored, wireless networks of smart sensing devices can be used to divide the monitoring area and analyze at the same time different portions of the plant [22,23]. Furthermore, smart sensor networks can allow the online implementation of algorithms for preprocessing the acquired data [24]. While complex classification algorithms based on convolutional neural networks like the one proposed in [5] cannot be implemented directly on the sensor node devices, simpler anomaly detection methods based on ML are feasibly implementable online. The latter solution could allow rapid detection of anomalies in voltage and current signals. Once the anomaly is detected in the sensor node that is installed in the grid, an algorithm using all the data stored in the cloud could start to analyze all the data and can specifically determine what kind of PQD was happening at that moment. This will ensure a prompt response of the operation maintenance crew, and thus maximizing safety and availability of the services.

The aim of this research article is to comprehensively demonstrate the entire process, the methodologies utilized, and the results that were achieved by employing various ML algorithms. These algorithms were specifically applied to predict the occurrence of anomalies (i.e., PQDs) that may arise across different scenarios. By detailing these aspects, the article seeks to provide a thorough understanding of how ML can be leveraged to enhance the detection and prediction of such anomalies in various contexts, with reference to the online real-time application of the algorithm directly onboard of small, low-complexity, low-cost sensing devices.

## 2. Materials and Methods

### 2.1. Data Source

An experimental dataset was created to provide a diverse set of signals that can be used for various analysis and research purposes related to electrical disturbances and anomalies, collecting different possible values by each phase. By including values of normal, not normal, and disturbance signals, the dataset allows the exploration and evaluation of different detection and classification algorithms applied specifically in the ML field. The normal signal values represent the desired and expected behavior of the power supply. These samples can serve as a baseline for comparison and analysis. On the other hand, the anomalies and disturbances are intentionally introduced to simulate real-world scenarios where electrical variances occur.

**Figure 1:**
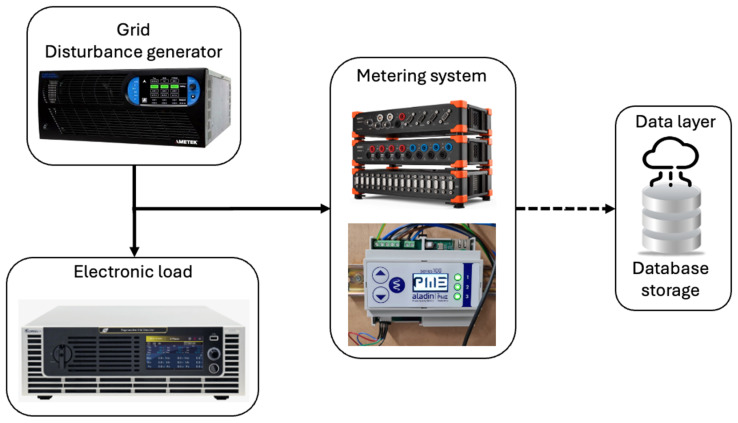
Schematic representation of the experimental setup used for the dataset generation.

The dataset was created using a programmable AC power source that was used to simulate the behavior of the grid. Indeed, it is possible to replicate disturbances inside the grid using such equipment, emulating a realistic scenario in a controlled environment. Using the electronic regenerative load, it was possible to simulate the behavior of different kinds of loads and scenarios, such as inductive or capacitive, distorted, and unbalanced loads. The monitoring and acquisition of the electrical power signal was done by dedicated instrumentation that allows for real-time data collection and analysis, helping to identify potential problems or issues in power systems and ensuring optimal performance and efficiency. A custom-made sensing device has been developed with the aim of measuring and recording voltage and current signals in a five-wire, three-phase system (i.e., phase L1, phase L2, phase L3, neutral wire, and ground wire). A deeper description of the custom measurement unit is provided in [25]. The correct monitoring of the electrical parameters using the proposed PQ meter could allow detection of unexpected failures and improve the energy efficiency of the power supplier. All the information captured by the different sensors in the devices was stored in a cloud-based database. The whole dataset is composed of a matrix of 64 columns by 28,000 rows, having a total size of 14.1 megabytes (Mb). This dataset could be considered a time series because it is composed of observations or data points collected sequentially over time. Each data point is associated with a specific timestamp, allowing for the analysis of trends, seasonal patterns, and correlations over time. A commercial PQ instrument by Dewesoft Sirius Modular Data Acquisition System (Dewesoft, Trbovlje, Slovenia) has also been used to check and validate the data acquired by the custom-made sensing device. The complete experimental platform used to generate the experimental datasets is shown in Figure 1.

**Figure 2:**
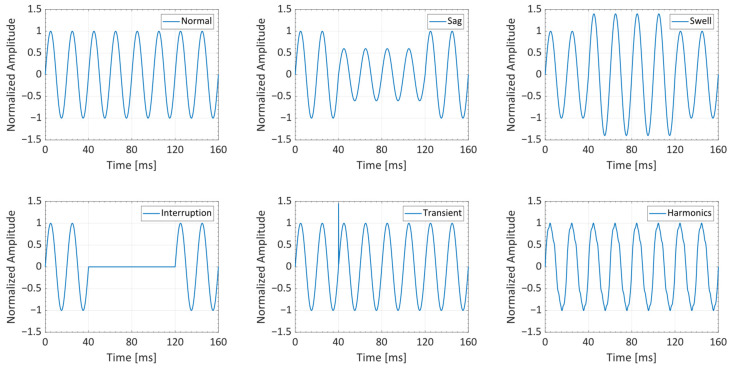
Graphical representation of the different PQDs considered in this work.

The disturbances that are included in the dataset are sag, swell, interruption, transient, and harmonics as defined in the IEEE recommended practice for monitoring electric PQ standard [4]. A sag is defined as a short duration decrease in voltage or current levels, typically lasting from a few cycles to a few seconds. It is defined as a decrease of voltage or current between 10% and 90% of the nominal voltage. Quite the opposite, a swell is a short increase (more than 10% of the nominal value) in voltage or current, lasting from a few cycles to a few seconds. A voltage interruption refers to a complete loss of voltage or current for a specified duration, typically lasting from a few milliseconds to several minutes. Transients are short, high-frequency voltage spikes or surges in electrical systems, typically lasting for a very short duration of microseconds to a few milliseconds. Harmonics happen in the presence of one or more sinusoidal harmonics at frequencies that are integer multiples of the fundamental frequency (typically 50 Hz or 60 Hz), which can distort the waveform. All the previous disturbances are shown in Figure 2.

### 2.2. Algorithm Pipeline

**Figure 3:**
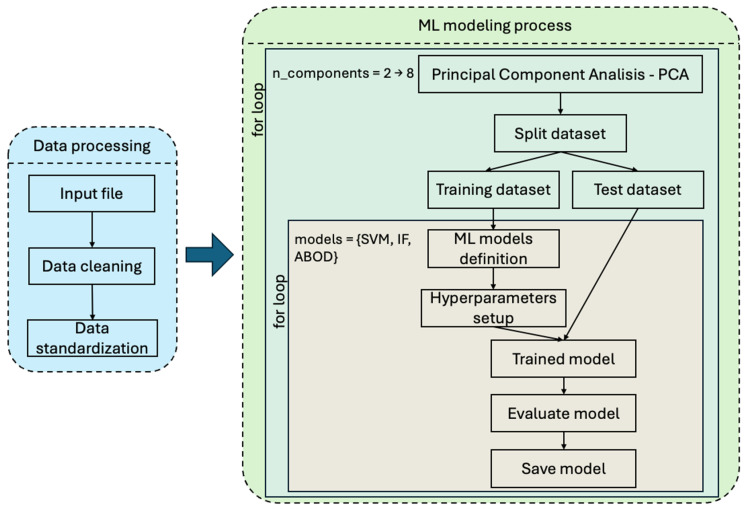
Pipeline of the ML algorithm’s implementation.

Once the dataset was captured and defined with all the PQDs, the experimental dataset was used as an input for the proposed ML algorithms. The further steps of the pipeline are the training and evaluation of the different models as illustrated in Figure 3. The input data set has 64 features. For each measurement sample, the feature map includes the datetime variable recording the acquisition time. Furthermore, for both voltage (V) and current (I) signals, the most common features are stored in dedicated fields related to every monitored conductor (three phases plus neutral). These include fields like the minimum Root Mean Squared (RMS), the maximum RMS, total energy, active power, apparent power, the first harmonic, total harmonic distortion, imbalance, and metadata of each device. Using the fields mentioned before, it is possible to identify the different PQDs previously shown in Figure 2.

The initial stage of the process focuses on data processing, which is illustrated in the left part of Figure 3. During this stage, the significant task of refining the dataset by eliminating certain columns that were unnecessary for the analytical objectives was executed, and the final dataset included 52 features. This step is crucial to enhancing the efficiency and clarity of the data that were used. Additionally, it was necessary to change the data types of several columns to ensure that they were appropriate for the operations we intended to perform. This transformation is vital for maintaining the integrity of the analysis and for facilitating seamless data manipulations thereafter. Finally, a reset of the index of the dataframe was executed, which is an important step to streamline the dataset and prepare it for subsequent processing and analysis.

Once the final features were defined, standardization was necessary. To implement this, we used the standard scaler technique. Basically, this transforms the data to have a mean (μ) of 0 and a standard deviation (σ) of 1. This process is often necessary because many ML algorithms perform better or converge faster when features are on a relatively similar scale and close to normally distributed. The following formulas show all the processes to obtain the final standardization for each column. The standard scores (z) of the samples are calculated as follows:

(1) $$z=\frac{x-\mu }{\sigma },$$

With mean defined in (2), where *N* is the number of samples, and $X_{i}$ is each individual feature value:

(2) $$\mu =\frac{1}{N}\sum_{i=1}^{N}\left(X_{i}\right),$$

The standard deviation calculation is detailed in (3).

(3) $$\sigma =\sqrt{\frac{1}{N}\sum_{i=1}^{N}{\left(X_{i}-\mu \right)}^{2}},$$

**Figure 4:**
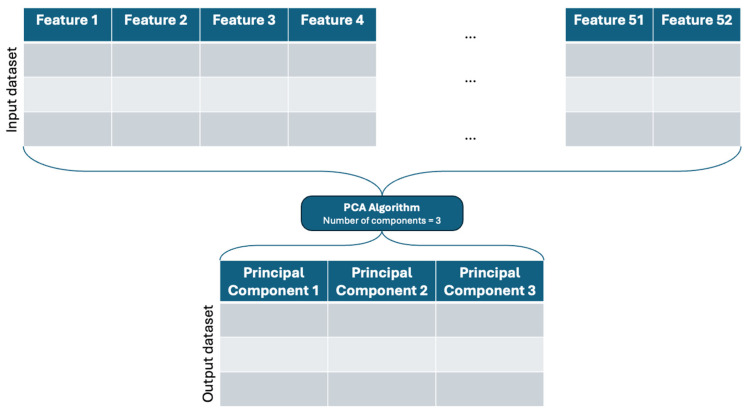
PCA’s algorithm graphic representation.

The result of the standardization step is the input of the Principal Component Analysis (PCA). The PCA technique was introduced by Harold Hotelling in [26]. Nowadays, PCA is a widely used statistical technique for dimensionality reduction and has various applications across numerous fields such as dsata visualization, face recognition, image compression, preprocessing for machine learning, and anomaly detection. This work is used as a dimensionality feature reduction, which simplifies the analysis and interpretation of the dataset. By transforming the data into a new coordinate system, PCA helps in identifying key patterns and features while reducing the number of dimensions without losing much information. The original dataset has 64 features, and in each feature is data of different types, such as string, datetime, integers, or decimals. The input dataset of the PCA algorithm is composed of 52 features, as was mentioned before in the explanation of the data processing pipeline, and is shown in Figure 4. This figure is reduced to 3 components for the graphic understanding, but it was tested during the training and testing phases using different numbers of components, as explained below.

**Figure 5:**
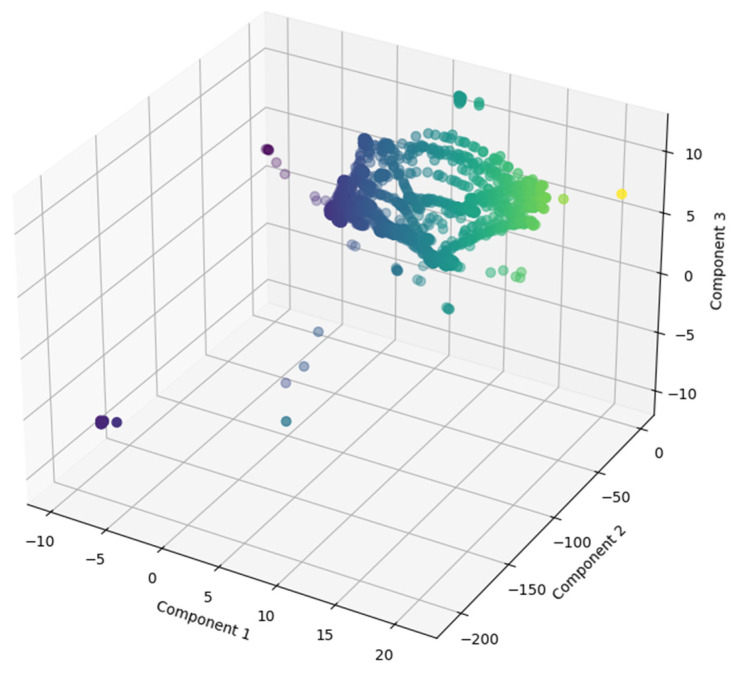
Three-dimensional representation of each component after applying the PCA reduction technique.

The dataset under study in this article is visually represented in Figure 5. As was said before, the original dataset, which comprised 52 distinct features, experienced a dimensionality reduction process through PCA, reducing the data from its initial 52 features into just 3 PCs. This significant reduction in dimensions enables a three-dimensional visualization of the dataset, which is important for enhancing comprehension of its inherent structure and behavioral patterns. In the first steps of the data analysis, it is helpful to present the data in this form because it is easier to identify relationships, trends, and crucial insights that might be obscured in a higher-dimensional space. Each principal PC is represented on its respective axis in this graph, making it easy to identify anomalies. This also demonstrates the effectiveness of the reduction technique used.

**Figure 6:**
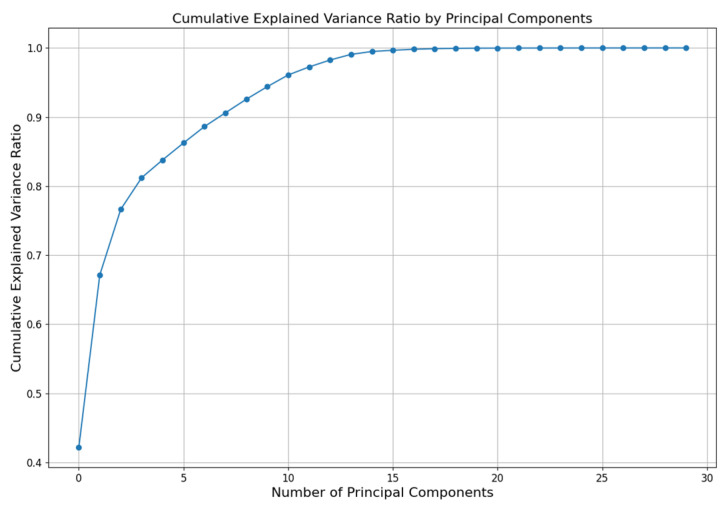
Cumulative explained variance ratio for the dataset used, applying the PCA algorithm.

One of the PCA’s algorithm hyperparameters is the number of Principal Components (PCs) to which the user wants to reduce the dimensionality of the input dataset. Each PC corresponds to a certain amount of variance in the data; this is called explained variance. The first PC captures the largest portion of the variance, the second captures the second largest portion, and so on. Also, it is important to know that each PC has an associated eigenvalue, which quantifies the amount of variance carried by that component. The eigenvalues are derived from the covariance matrix of the data. Figure 6 graphically represents the behavior of the variance ratio applied to our dataset. In this figure, it is possible to appreciate that, when reduced to 15 PCs, the explanation of the dataset was totally included without any loss of information. This graph displays the proportion of the total variance explained by each PC. The *x*-axis represents the principal components, while the *y*-axis shows the variance ratio (eigenvalues) for each component. A key aspect of interpreting this graph is identifying the “elbow”, where the amount of additional variance explained by subsequent components begins to level off. This point indicates the optimal number of principal components to retain for capturing most of the variance in the data, balancing dimensionality reduction with information retention. A sharp drop in variance after certain components suggests that those primary components capture most of the essential information in the dataset. Also, in this figure it is possible to see the cumulative explained variance ratio, which is the running total of the explained variances. Basically, it gives a holistic view of how much of the total variance is explained, including more PCs. In this research, the number of PCs went from 2 up to 8 PCs in the output. With 2 PCs, it explains almost 80% of the data, with 3 it explains 82%, and so on. 8 PCs explain almost 92%, so the algorithm iterates over these PCs to get the best results with the developed experimental dataset.

Using the prepared dataset with the PCs processed in the last step, the dataset is split in two parts: the training and test sets. The training set is used to teach the model, while the test set assesses how well the model generalizes to new, unseen data, helping to detect overfitting and ensuring the model’s robustness.

The first ML algorithm tested was One Class Support Vector Machine (OCSVM), presented in this paper for the first time [27]. It is specifically designed for anomaly detection. It works by creating a boundary around the normal data points in a feature space, effectively defining a hypersphere that encompasses most of the training data. The algorithm identifies this boundary by maximizing the margin between the normal data points and the origin while allowing for a certain number of outliers. During the training phase, it finds the optimal parameters that best separate these points, using a kernel function to handle non-linear relationships. When new data points are introduced, they are classified as anomalies if they fall outside this learned boundary, making OCSVM particularly effective for situations where only normal instances are available for training.

The second ML algorithm is the Isolation Forest (IF), an anomaly detection algorithm that works by isolating observations in a dataset to identify anomalies introduced in [28]. It builds multiple decision trees, known as isolation trees, by randomly selecting a feature and then randomly selecting a split value to partition the data. Anomalies, which are typically few and different from most of the data, tend to be isolated quickly by these trees, resulting in shorter path lengths. On the other hand, normal observations require more splits to isolate, leading to longer path lengths. The algorithm computes an anomaly score based on the average path length across all trees, with shorter path lengths indicating a higher likelihood of being an anomaly.

The last tested algorithm is Angle-Based Outlier Detection (ABOD), an anomaly detection technique that assesses the behavior of data points based on the angles they form with pairs of other points in the dataset. Specifically, ABOD calculates the variance of the angles formed by a target point with its neighboring points; a high variance indicates that the target point is positioned differently compared to others, suggesting it may be an outlier well explained in [29]. This method is particularly effective in high-dimensional spaces, as it captures relationships between points that might be overlooked by distance-based methods. By focusing on angular relationships rather than just distances, ABOD can detect anomalies that may be close to normal points in terms of traditional metrics but are structurally distinct.

## 3. Results and Discussion

In the process of generating the dataset utilized for both the training and testing phases, a careful identification of the records was undertaken. This involved distinguishing between those records that exhibited what was classified as normal behavior and those that contained instances of disturbances. By organizing the data in this manner, the goal was to streamline the calculation of various classification performance metrics and enhance the evaluation process of the different models employed. This systematic approach allowed for a clearer understanding of how well each model performed under varying conditions, ultimately contributing to more reliable and informed conclusions about their effectiveness.

### 3.1. Metrics

This subsection delves into the various metrics employed to evaluate the performance of ML anomaly detection algorithms. We explored key statistical measures such as precision, recall, accuracy, F1 score, Receiver Operating Characteristic (ROC), Area Under the Curve of ROC (AUC-ROC), and the confusion matrix, as are clearly explained in [30]. These metrics provide insights into the trade-offs between false positives and false negatives, ultimately guiding the optimization process for different applications. By rigorously analyzing these metrics, it is easy to understand and paint a clear picture of the strengths and weaknesses of the employed algorithms, fostering a deeper understanding of their efficacy in real-world scenarios. Understanding and applying these metrics enables better decision-making regarding algorithm selection and tuning the hyperparameters.

#### 3.1.1. Confusion Matrix

This is a table that summarizes the results of a classification problem by providing a breakdown of the predicted versus actual classifications for a given dataset. Typically, it consists of four key components: True Positive (TP), False Positive (FP), True Negative (TN), and False Negative (FN).

- **TP** refers to the cases where the model correctly predicts the positive class.
- **FP** indicates the instances where the model incorrectly predicts the positive class.
- **TN** captures the scenarios where the model accurately identifies the negative class.
- **FN** represents cases where the model fails to identify the positive class.

This matrix is especially useful for classification problems, as it can provide insights into not only the overall effectiveness of the model but also its performance on individual classes, facilitating a deeper understanding of potential biases or areas for improvement in the model’s predictive capabilities.

#### 3.1.2. Precision

This is a metric used to evaluate the accuracy of a classification model, particularly in scenarios where the costs of false positives are high. It is defined as the ratio of true positive predictions to the sum of true positive and false positive predictions. Mathematically, precision can be expressed by Formula (4):

(4) $$Precision=\frac{TP}{TP+FP}\;,$$

A higher precision value indicates a lower rate of FP, making the model more reliable in its positive predictions. This metric is especially valuable in imbalanced datasets where one class significantly outnumbers the other, helping to highlight the performance of the model in correctly identifying the minority class.

#### 3.1.3. Recall

This is a metric used more in scenarios where identifying positive instances is important. It is also known as sensitivity or TP rate, and measures the proportion of actual positive cases that were correctly identified by the model. It can be expressed by Formula (5).

(5) $$Recall=\frac{TP}{TP+FN},$$

Given an interpretation of this metric, a high recall indicates that the model successfully captures most of the positive cases. This could be important depending on the context of the algorithm’s application and the business case scenarios of test and use.

#### 3.1.4. Accuracy

This measures how well a classification model predicts the correct labels for a given dataset. It is defined as the ratio of the number of correct predictions to the total number of predictions made, as shown in Formula (6).

(6) $$Accuracy=\frac{TN+TP}{TN+FP+TP+FN},$$

This metric provides a straightforward measure of a model’s overall ability to correctly classify the data, but it can be misleading in cases of imbalanced datasets, where one class significantly outnumbers the others, and it is necessary to use other metrics to make well-rounded decisions in assessing model performance.

#### 3.1.5. F1 Score

This is the harmonic mean of precision and recall, providing a single score that balances both FP and FN, well explained previously. The formula for the F1 score is given by (7):

(7) $$F1\;Score=2\cdot \frac{Precision\cdot Recall}{Precision+Recall}\;,$$

This formula highlights that the F1 score will only be high if both precision and recall are high, making it especially useful in contexts where false negatives are costly or detrimental.

#### 3.1.6. Receiver Operating Characteristic—ROC

This is a graphical representation used to evaluate the performance of binary classification models by plotting the TP Rate (TPR) against the FP Rate (FPR) at various threshold settings. The TPR, called sensitivity or recall, is defined as the ratio of correctly predicted positive observations to the actual positives. The FPR, on the other hand, is the ratio of incorrectly predicted positive observations to the actual negatives. The ROC curve allows practitioners to visualize the trade-off between sensitivity and specificity across different classification thresholds, and the Area Under the ROC Curve (AUC-ROC) serves as a single scalar value summary of the model’s performance, with values closer to 1 indicating better predictive performance.

#### 3.1.7. Discussion about the Metrics Useful for the Specific Case Study

Overall, in scenarios where missing a positive instance has significant consequences, the recall metric should be emphasized. A high recall indicates that most positive cases are correctly identified; also, this is an important metric in scenarios where the cost of false negatives is high. Both of those considerations applied in the case of this study. As a matter of fact, the main goal of the algorithm is to detect all occurrences of PQDs, that is, to identify the largest number of positive cases correctly, trying to prevent problems on the grid and optimizing the quality of the power suppliers.

On the other hand, the F1 score allows for a comparative assessment that takes both precision and recall into account, making it easier to identify which model performs overall better in identifying anomalies. By simultaneously considering FP and FN, it helps to understand the model’s effectiveness in identifying rare events, which is essential for reliable decision-making in this field of study.

### 3.2. Outcomes

As previously mentioned, each model utilized in this work has its own specific set of hyperparameters that were determined and predefined prior to the initiation of the code execution process. Also, it is important to note that the number of PCs used in the analysis serves as a crucial hyperparameter. This hyperparameter can be systematically varied and tested, with the goal of optimizing the outcomes derived from each distinct model being evaluated. By iterating through different values for the number of principal components, it assesses how changes influence the model’s performance and accuracy, thereby allowing identification of configurations that yield superior results relative to the specific objectives of the analysis.

The primary objective behind establishing these hyperparameters is to create a framework that allows for the exploration of a diverse range of variables, each represented by a unique combination of values during every run of each model. This strategic approach is designed to enhance several critical aspects of the modeling process, including the learning rate, execution time, overall performance, and the maximization of key performance metrics. By systematically varying the combinations of hyperparameter values, the aim is to identify the optimal settings that lead to improved outcomes, enabling the model to learn more effectively and efficiently from the data it processes. For the OCSVM model, a total of 80 different combinations have been tested, while 120 different possible options were considered for IF and ABOD. All those possible combinations were made by changing the values of each hyperparameter; once one value is changed, this gives a new combination to test in each algorithm. All the possible hyperparameters for each algorithm are shown in Table 1. Also, the top three models with the best recall of all the possible combinations are presented for each model. In general, the best models are derived from the OCSVM algorithm, achieving perfect classification of all disturbances in the test dataset. One important point to note is that all models achieving a recall of 1 were tested with the same hyperparameters; the only variable that changed among them was the number of components in the input dataset. Finally, analyzing the other metrics, the second model appears as the best with a precision, F1 score, and accuracy of 0.6, 0.75, and 0.666, respectively, yielding the highest results using 4 PCs.

**Figure 7:**
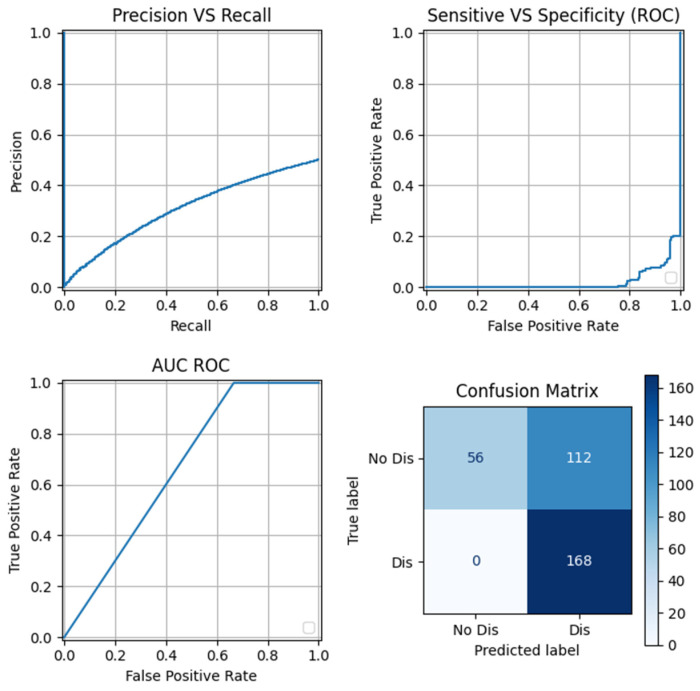
Set of graphs showing the best model according to the recall metric.

Figure 7 graphically presents some of the metrics explained in Section 3.1 which correspond to the best model according to Table 1, which is the second row of the OCSVM model. The first graph from the top left to the right down is the “Precision vs. Recall” graph, which visualizes the trade-off between precision and recall for a model across various thresholds. This visualization is helpful in understanding the balance between identifying TP and avoiding FP, guiding them in selecting an appropriate threshold based on their specific objectives and the implications of FP and FN in their application. Analyzing in detail the confusion matrix for the best model, all the disturbances were identified correctly. In this case of study, is better to identify all of them in order to minimize problems in the grid and the impact that could have on the industry.

Table 2 displays the top three models with the highest F1 scores among all tested combinations for each model. Also, in this case, the OCSVM algorithm achieved the best metrics, with the F1 score reaching up to 92% and accuracy up to 90%, also with the dataset reduced to 4 PCs. The second-best algorithm, which showed the best balance, is ABOD, achieving F1 scores up to 91%.

**Figure 8:**
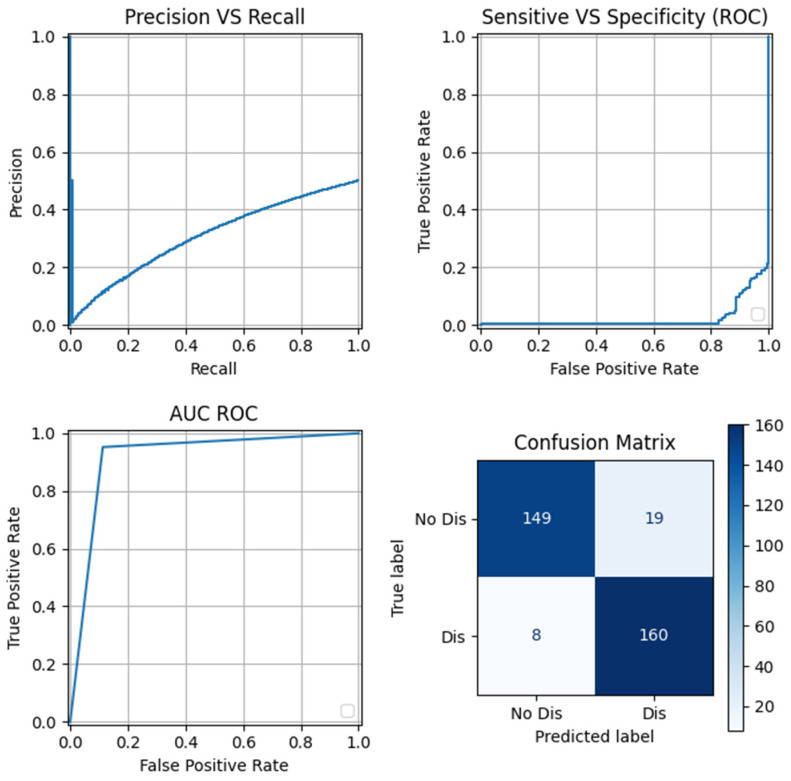
Collection of graphs for the best model based on the F1 score metric.

Figure 8 visually illustrates the different graphs that correspond to the best model outlined in Table 2, which is the first row of the OCSVM model. The evaluation of the F1 score in this case of study is important to identify true anomalies while minimizing FP and FN, because one anomaly that is not identified correctly may occur in equipment malfunctions or failures, leading to costly repairs and unplanned downtime for industrial processes. Sensitive electronics and machinery can experience operational disruptions or even permanent damage, affecting productivity and safety.

Additionally, it can strain power systems, resulting in inefficiencies and increased energy costs. Over time, the cumulative effect of these disturbances can severely impact electrical infrastructure, reduce the lifespan of devices, and lead to significant financial losses for businesses. Furthermore, the failure to detect these issues can compromise compliance with regulatory standards, potentially resulting in legal repercussions and further financial penalties.

**Figure 9:**
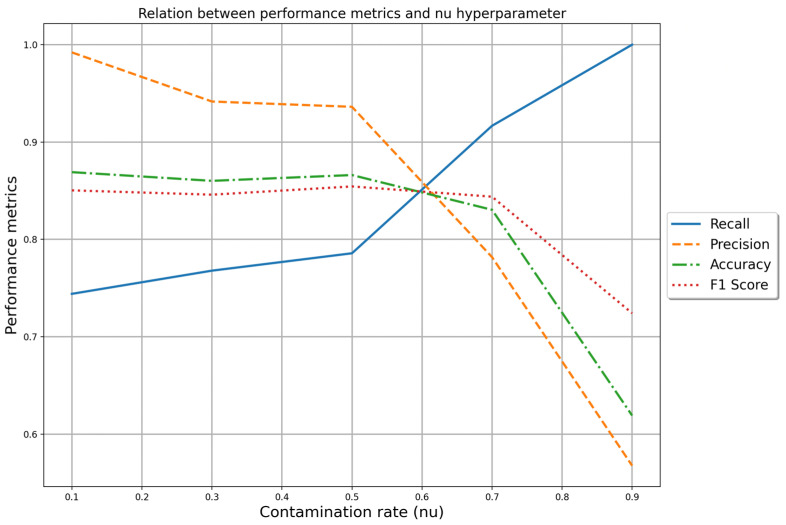
Relationship between the performance metrics and the “nu” hyperparameter for the best model OCSVM.

After the execution of all scenarios was performed, an analysis of the results from the best model, OCSVM, revealed a relationship between the “nu” hyperparameter and the performance metrics, as shown in Figure 9. The “nu” hyperparameter serves as a regularization term that balances the trade-off between the model’s complexity and its ability to identify outliers. Specifically, “nu” defines an upper bound on the fraction of training errors (points that do not belong to the target class) and a lower bound on the fraction of support vectors used in the model. The relation was that when the “nu” increases, the recall metric also increases, but the other metrics start to decrease. It happens when the decision threshold of your model is adjusted to increase recall (i.e., you make the model more compliant in predicting positive instances), and you often capture more TP.

However, this can lead to more FP as well, which would decrease precision since more incorrect positive predictions reduce the precision score. Since the F1 score is a balance between precision and recall, when precision drops significantly while recall is increasing, the F1 score will also decrease.

## 4. Conclusions

This study successfully demonstrates the development and testing of novel ML models that identify anomalies in smart grids, complex systems, and big plants. These models can be implemented directly on the sensor nodes used for signal acquisition, providing to the end users a tool that notifies and advises them when an anomaly occurs in the power supply system’s quality.

The results indicate that the OCSVM model is the most effective at identifying anomalies accurately. It was evaluated using various proposed techniques applied to the different options tested while iterating through the hyperparameters. Those models achieved a recall of 100% and an F1 score of 92%.

The various tested models can easily adapt to the volume of data generated by different sensors during the collection process, making them suitable for scaling in any industry. The dataset may contain numerous features, and the preprocessing technique effectively reduces the data to a specific number of principal components without losing valuable information from the original dataset.

## Figures and Tables

**Table 1:** Top three metrics from each tested model, based on recall.

Model	N Components	Hyperparameters Tested	Recall	Precision	F1 Score	Accuracy
One Class Support Vector Machine OCSVM	3	nu = 0.9; kernel = rbf; gamma = scale	1.0	0.5833	0.7368	0.6428
	4	nu = 0.9; kernel = rbf; gamma = scale	1.0	0.6	0.75	0.6666
	5	nu = 0.9; kernel = rbf; gamma = scale	1.0	0.5676	0.7241	0.6191
Isolation Forest IF	9	estimator = 100; contamination = 0.5	0.9166	0.5256	0.6681	0.5446
	9	estimator = 10; contamination = 0.5	0.9048	0.5333	0.6711	0.5565
	3	estimator = 50; contamination = 0.5	0.8988	0.9438	0.9207	0.9226
Angle-Based Outlier Detection ABOD	8	contamination = 0.5; neighbors = 5	0.9881	0.5355	0.6946	0.5655
	9	contamination = 0.5; neighbors = 5	0.9881	0.4985	0.6627	0.4971
	7	contamination = 0.5; neighbors = 10	0.9821	0.5830	0.7317	0.6399

**Table 2:** The top three metrics from each evaluated model, ranked by F1 score.

Model	N Components	Hyperparameters Tested	Recall	Precision	F1 Score	Accuracy
One Class Support Vector Machine OCSVM	4	nu = 0.7; kernel = rbf; gamma = scale	0.9524	0.8939	0.9222	0.9196
	4	nu = 0.5; kernel = rbf; gamma = auto	0.8988	0.9321	0.9152	0.9167
	3	nu = 0.7; kernel = rbf; gamma = scale	0.9345	0.8870	0.9101	0.9078
Isolation Forest IF	3	estimator = 50; contamination = 0.5	0.8988	0.9438	0.9207	0.9226
	4	estimator = 50; contamination = 0.5	0.8393	0.9463	0.8896	0.8958
	4	estimator = 100; contamination = 0.5	0.8214	0.9650	0.8875	0.8958
Angle-Based Outlier Detection ABOD	4	contamination = 0.1; neighbors = 10	0.8869	0.9551	0.9198	0.9226
	4	contamination = 0.1; neighbors = 5	0.8809	0.9610	0.9193	0.9227
	3	contamination = 0.1; neighbors = 5	0.8809	0.9548	0.9164	0.9196

## Data Availability

The raw data supporting the conclusions of this article will be made available by the authors on request.
